# Pre-eclampsia among Pregnant Women Admitted to the Department of Obstetrics and Gynaecology of a Tertiary Care Centre

**DOI:** 10.31729/jnma.8265

**Published:** 2023-11-30

**Authors:** Narayan Mahotra, Sonam Chaudhary, Pooja Poudyal

**Affiliations:** 1 Department of Clinical Physiology, Maharajgunj Medical Campus, Maharajgunj, Kathmandu, Nepal; 2 Department of Gynecology and Obstetrics, Maharajgunj Medical Campus, Maharajgunj, Kathmandu, Nepal

**Keywords:** *maternity*, *Nepal*, *pre-eclampsia*, *prevalence*

## Abstract

**Introduction:**

Pre-eclampsia is a pregnancy-related hypertensive disorder with maternal and neonatal complications. Many studies are done regarding the prevalence of pre-eclampsia in Nepal but ascertaining the maternal risk factors and fetal outcomes are important. The aim of this study was to find out the prevalence of pre-eclampsia among pregnant women admitted to the Department of Obstetrics and Gynaecology of a tertiary care centre.

**Methods:**

A descriptive cross-sectional study was conducted among pregnant women admitted to the Department of Obstetrics and Gynaecology of a tertiary care hospital from 13 July 2023 to 29 September 2023 after obtaining ethical approval from the Institutional Review Committee. Convenience sampling method was used. Point estimate was calculated at a 95% Confidence Interval.

**Results:**

Among 5065 patient, pre-eclampsia was seen in 44 (0.87%) (0.61-1.13, 95% Confidence Interval). A total of 16 (36.36%) cases of pre-eclampsia were in the age group 25-29 years and 30 (68.18%) of them were nulliparous. A total of 38 (86.36%) of the newborns of the pre-eclamptic cases had low birth weight. The APGAR score of newborns at the 1 minute after birth was 26 (59.09%) followed by the newborns who needed re-evaluation 16 (36.36%). The APGAR score recorded at 5 minutes showed maximum newborns with normal APGAR score 40 (90.90%).

**Conclusions:**

The prevalence of pre-eclampsia among pregnant women was found to be lower than other studies done in similar settings.

## INTRODUCTION

Pre-eclampsia is a pregnancy-related hypertensive disorder marked by the onset of hypertension and proteinuria in a previously normotensive and non-proteinuric pregnant woman after the 20^th^ week of gestation.^1^ It can lead to abortion, prematurity, intrauterine growth retardation and stillbirth in the case of a fetus,^2^ causing maternal, perinatal and neonatal morbidity and mortality.

Around 5-10% of all pregnancies are affected by preeclampsia with the increase in incidence in developing countries.^3^ The extremes of maternal age and nulliparity are considered to be important risk factors for the development of this condition.^4^ This condition of pre-eclampsia is associated with poor pregnancy outcomes affecting fetal viability, birth weight and

APGAR scores.^5^ This brings the importance of re-ascertaining the risk factors in pre-eclampsia and its impact on the fetus.

This study aimed to find out the prevalence of preeclampsia among pregnant women admitted to the Department of Obstetrics and Gynaecology of a tertiary care centre.

## METHODS

A descriptive cross-sectional study was conducted among pregnant women admitted to the Department of Obstetrics and Gynaecology of Tribhuwan University Teaching Hospital, Maharajgunj, Kathmandu, Nepal from July 2023 to September 2023. The non-digitalised hospital records of maternity cases within a duration of 17 months from April 2022 onwards were obtained from the hospital record section after getting ethical approval from the Institutional Review Committee (Reference number: 07(6-11)E-2 080/081). The maternity cases of Nepali origin with no missing information were included. A convenience sampling method was used. The sample size was calculated with the following formula.


$$\begin{array}{ll} \text{n} & ={\text{Z}}^{2}\times \frac{\text{p}\times \text{q}}{{\text{e}}^{\text{2}}}\\ & =1.96^{2}\times \frac{0.5\times 0.5}{0.02^{2}}\\ & =2401 \end{array}$$

Where,

n = minimum required sample size
Z = 1.96 at 95% Confidence Interval (CI)
p = prevalence taken as 50% for maximum sample size calculation
q = 1-p
e = margin of error, 2%

The calculated minimum required sample size was 2401. After doubling, sample of 4802 was calculated. However, a total of 5065 maternity cases were included in the study.

The study variables were maternal age and parity as risk factors for pre-eclampsia and the fetal viability, birth weight and APGAR scores were considered as fetal outcomes. The parity among the cases was again classified as nulliparous, primiparous and multiparous. The fetal viability was classified as dead or alive newborns; the birth weight of newborns was classified as low birth weight (<2.5 kg), high birth weight (>4 kg) and in between as normal birth weight. The APGAR score recorded was again classified into 3 categories 7-10 (normal), 4-6 (needs re-evaluation) and 0-3 (never good requiring immediate attention).^6^ The appearance, pulse, grimace, activity and respiratory parameters were scored at 1 and 5 minutes respectively after birth.

Data were entered in Microsoft Excel 2016 and analysed using IBM-SPSS Statistics version 16.0. Point estimate was calculated at a 95% CI were calculated.

## RESULTS

Among 5065 pregnant women, the distribution of pre-eclampsia was 44 (0.87%) (0.61-1.12, 95% CI). The mean age among the cases of pre-eclampsia was 30.70±5.09 years. A total of 16 (36.36%) cases were between 25-29 years, 11 (25%) between 35-39 years and only 2 (4.54%) were between 40-44 years. A total of 30 (68.18%) of the cases were nulliparous and 11 (25%) were primiparous (Table 1).

**Table 1:** Distribution of maternal risk factors among the cases of pre-eclampsia (n= 44).

Maternal risk factors	
Age (years)	n (%)
20-24	5 (11.36)
25-29	16 (36.36)
30-34	10 (22.72)
35-39	11 (25.00)
40-44	2 (4.54)
Parity	
Nulliparous	30 (68.18)
Primiparous	11 (25.00)
Multiparous	3 (6.81)

All of the newborns of the pre-eclamptic cases were alive. The mean birth weight of the newborns was 1.84±0.69 years. A total of 38 (86.36%) of the newborns had low birth weight and 6 (13.63%) had normal birth weight. The APGAR score was studied in the first and 5 minutes after birth. A total of 26 (59.09%) of the newborns had APGAR scores between 7-10, 16 (36.36%) had between 4-6 and 2 (4.54%) had between 0-3 in 1 minute after birth. A total of 40 (90.90%) newborns had APGAR scores in between 7-10 and 4 (9.09%) scored between 4-6 in the 5 minute after birth (Table 2).

**Table 2:** Distribution of newborn outcomes among the cases of pre-eclampsia (n= 44).

Newborn outcomes	
Fetal viability	n (%)
Alive	44 (100)
Dead	-
**Birth weight**	
Low birth weight	38 (86.36)
Normal birth weight	6 (13.63)
High birth weight	-
**APGAR score (1 minute)**	
7-10	26 (59.09)
4-6	16 (36.36)
0-3	2 (4.54)
**APGAR score (5 minutes)**	
7-10	40 (90.90)
4-6	4 (9.09)
0-3	-

## DISCUSSION

Pre-eclampsia is associated with maternal, perinatal and neonatal morbidity and mortality with increased incidence of the disease in developing countries.^3^ In our sudy, among 5061 pregnant women, 44 (0.87%) had pre-eclampsia which is similar to the study done in Nepal.^7^

This study has found that the distribution of the cases was maximum in age group 25-29 (36.36%). This finding was similar to another retrospective study conducted in Nepal which has also found the highest prevalence of pre-eclampsia in the normal reproductive age group (83.52%). Also, 69.6% of cases of preeclampsia belonged to the age group 20-34 years in a cross-sectional study conducted in Malaysia.^5^ The finding was similar among the pre-eclamptic cases in Uganda as well.^8^ This age group is the most common age range for marriage and family planning among women which is supported by the highest rate of pregnancy and its associated complications like preeclampsia in this age.

This study has also found that 68.18% of pre-eclamptic cases were nulliparous, followed by primiparity (25%) and multiparity (6.81%). The nulliparity is also considered an important risk factor for developing the condition.^4^ A similar study done in Saudi Arabia has found that the highest proportion of pre-eclamptic cases (42.0%) were nulliparous.^9^ The finding was in contrast among the pre-eclamptic cases of Indonesia which reported the distribution of multiparous women being more in the condition.^10^

As per the outcomes among newborns considered in our study, all of them were alive 44 (100%), and 86.36% had low birth weight. A total of 26 (59.09%) had good APGAR scores in 1 minute which increased to 90.90% at 5 minutes. Studies have found that severe pre-eclampsia represents a significant risk factor for intrauterine fetal demise, with an estimated stillbirth rate being 21 per 1000.^11^ It also reported that the risk of fetal demise is over 50% less in cases of mild preeclampsia when compared with severe pre-eclampsia.^11^ The prevalence of stillbirth was 2.34% among the cases of pre-eclampsia.^9^ The frequency seemed to increase in the study conducted in Uganda with 12.6% cases of stillbirth in pre-eclamptic women.^8^

Studies have found that the mean birth weight of babies delivered by mothers who had early onset preeclampsia was lower than those of non-pre-eclamptic across all gestational ages.^12^ It supports the hypothesis of placental hypoperfusion due to shallow invasion of fetal trophoblast in pregnancy leading to fetal growth restriction in pre-eclampsia.^13^ The finding is similar to our study where the highest distribution of low-birth-weight babies was seen among the pre-eclamptic cases.

Studies have found that pre-eclamptic women have infants with lower APGAR scores than healthy women.^14^ However, similar studies done among pre-eclamptic cases in Indonesia have found that the neonates with normal APGAR score (>7/10) were highly distributed at both 1-minute (80.7%) and 5-minute intervals (94.6%).^10^ This finding was consistent with the result of our study which reported a higher normal APGAR score at both 1 and 5 minutes in the cases of pre-eclampsia.

The limitations of the study are minimum maternal risk factors and newborn outcomes were considered. Thus, a similar study considering various other maternal risk factors such as obesity, blood pressure, blood sugar level, antenatal care facility or any renal or cardiac diseases and fetal outcomes like the fetal size and proportions; haematological and other systemic effects in a larger sample size can be conducted.

## CONCLUSIONS

The prevalence of pre-eclampsia among pregnant women is lesser than in other similar studies done in similar settings.
